# Coefficient of Linear Thermal Expansion of Polymers and Polymer Composites: A Comprehensive Review

**DOI:** 10.3390/polym17233097

**Published:** 2025-11-21

**Authors:** Alexander G. Khina, Denis P. Bulkatov, Ivan P. Storozhuk, Alexander P. Sokolov

**Affiliations:** Bauman Moscow State Technical University, Moscow 105005, Russia; bulkatov@bmstu.ru (D.P.B.); storozhukip@bmstu.ru (I.P.S.); alsokolo@bmstu.ru (A.P.S.)

**Keywords:** coefficient of linear thermal expansion (CLTE), polymers, thermoplastics, thermosets, composites, polymer composite materials (PCMs), dimensional stability, dilatometry, thermomechanical analysis (TMA), X-ray diffractometry (XRD)

## Abstract

This work presents a comprehensive literature review of the coefficient of linear thermal expansion (CLTE) of polymers and polymer composite materials (PCMs). It systematizes CLTE measurement methods for isotropic and anisotropic materials, including contact techniques such as dilatometry and thermomechanical analysis and non-contact methods such as digital image correlation, laser interferometry, diffraction-based techniques, and strain-gauge methods, with attention to their accuracy and fields of applicability. Furthermore, the review describes the principal mathemaical modeling approaches used to predict the CLTE of polymers and PCMs. The review also provides a comparative analysis of CLTE values for a broad range of thermoplastics (commodity, engineering, and high-performance grades) and thermosets, identifying the key factors that govern CLTE, such as the transition from the glassy to the viscous-flow state, the presence and anisotropy of a crystalline phase, and related structure–property effects. Special consideration is given to the factors determining the CLTE of polymer composites, including the properties of the polymer matrix, the nature, size, orientation and surface treatment of the filler, the architecture and reinforcement scheme of the composite, and the manufacturing process. The review also outlines application areas in which PCMs with controlled or reduced CLTE are required and illustrates these with specific examples. Thus, the article provides integrated view of the CLTE of polymers and PCMs, compiles reference data for CLTE values of various polymers and common composite fillers and offers practical recommendations for selecting polymer materials for fabricating goods that require high thermal dimensional stability.

## 1. Introduction

One of the fundamental characteristics of materials of diverse physicochemical nature is the coefficient of thermal expansion (CTE), which is subdivided into the coefficients of linear and volumetric thermal expansion (CLTE and CVTE, respectively) [1,2,3]. The importance of these parameters arises from practical applications, in which materials are used in engineered components and structures and temperature-dependent dimensional changes must be taken into account [4,5].

The expansion of solids upon heating arises from an increase in the amplitudes of atomic vibrations and in the average interatomic distance [6,7]. The increase in a body’s volume upon heating is called volumetric expansion [8]. Volumetric expansion is characterized by the CVTE, denoted β, which quantifies the relative increase in volume per 1 °C rise in temperature:

(1) $$\beta =\frac{V-V_{0}}{V_{0}\left(T-T_{0}\right)}=\frac{∆V}{V_{0}∆T};$$

where *T*_0_, *T* are the initial and final temperatures (°C), respectively; *V*_0_ and *V* are the initial and final volumes of the specimen at *T*_0_ and *T*, respectively; Δ*V* is the change in volume; Δ*T* is the change in temperature.

It follows that

(2) $$V_{T}=V_{0}\left(1+\beta \Delta T\right)$$

The increase in length of isotropic solids upon heating, using the linear model of thermal deformation $\frac{∆l}{l_{0}}$ versus temperature change ∆T, is characterized by a scalar—the coefficient of linear thermal expansion (CLTE), denoted α—a scalar that specifies the relative change in any linear dimension (length, width, or thickness) per 1 °C rise in temperature:

(3) $$\alpha =\frac{l-l_{0}}{l_{0}\left(T-T_{0}\right)}=\frac{∆l}{l_{0}∆T}=\frac{\epsilon }{∆T};$$

where *l*_0_, *l* are the initial and final specimen lengths along the measurement direction at *T*_0_ and *T*, respectively; Δ*l* is the change in length along the measurement direction; and $\epsilon =\frac{∆l}{l_{0}}$ is the thermal deformation.

For small deformations in isotropic solids, the coefficient of volumetric thermal expansion equals three times the coefficient of linear thermal expansion, i.e., β = 3α [9,10]. For heterogeneous materials, including composite materials (CMs), it is typical that their physico-mechanical properties and, in particular, their thermal expansion characteristics differ with direction (anisotropy) depending on the microstructure, and therefore their CLTE can vary when measured along different directions [11,12]. For example, in anisotropic crystals, wood, fiber-reinforced composites, and many other materials, the CLTEs along three mutually perpendicular axes are different.

For anisotropic materials, for small linear deformations [13]:

(4) $$\beta =\alpha_{11}+\alpha_{22}+\alpha_{33}$$

where $\alpha_{11}$, $\alpha_{22}$, $\alpha_{33}$ are the CLTE along the material’s principal symmetry axes.

In the general three-dimensional anisotropic case, the CTEs depend on temperature according to the following relation:

(5) $$\begin{array}{l} \alpha_{ij}\left(T\right)=\;\frac{\partial \epsilon_{ij}}{\partial T} \end{array}$$

where $\epsilon_{ij}=\epsilon_{ij}\left(T\right)$ are the components of the thermal deformation tensor, and $\alpha_{ij}$ are the components of the second-rank thermal expansion tensor α.

In three-dimensional space $R^{3}$ the components $\alpha_{ij}$ are represented by a symmetric ${\left\|\alpha_{ij}\right\|}_{3\times 3}$ matrix (Table 1). The diagonal components, $\alpha_{xx}$, $\alpha_{yy}$, and $\alpha_{zz}$, represent the linear thermal expansion coefficients along their respective coordinate axes, indicating the material’s elongation or contraction per 1 °C temperature increase. The off-diagonal components $\alpha_{xy}$, $\alpha_{xz}$, $\alpha_{yz}$ likewise characterize the relation between a temperature change and the shear (angular) deformation. In particular cases, the number of independent components of the tensor α varies (Table 1).

By integrating (5) over temperature on the interval $\left[T_{0},T\right]$, the following expression is obtained:

(6) $$\epsilon_{ij}\left(T\right)-\epsilon_{ij}\left(T_{0}\right)=\int_{T_{0}}^{T}\alpha_{ij}\left(\tau \right)d\tau ={\bar{\alpha }}_{ij}∆T$$

where τ is the integration variable, ∆T is the temperature change relative to the reference temperature $T_{0}$, for which $\epsilon_{ij}\left(T_{0}\right)=0$ is assumed, and ${\bar{\alpha }}_{ij}$ are the components of the thermal expansion tensor averaged over the temperature interval $\left[T_{0},T\right]$:

(7) $${\bar{\alpha }}_{ij}={\left\langle \alpha_{ij}\left(\tau \right)\right\rangle }_{\left[T_{0},T\right]}=\frac{1}{\Delta T}\int_{T_{0}}^{T}\alpha_{ij}\left(\tau \right)d\tau .$$

For small temperature changes, the corresponding deformations are determined by the tensor of small thermal deformations, which depends linearly on the temperature increment:

(8) ϵ=αΔT

where α is a constant CLTE tensor with components $\alpha_{ij}$, which are temperature-independent constants.

The CLTE is expressed in K^−1^ (or °C^−1^) units of measurement, but due to the small values of the CLTE it is often expressed in ppm/°C (parts per million per °C) or μm/(m·K).

For many solids, the CLTE has small values—typically on the order of 10^−6^–10^−5^ K^−1^ (or 1–10 ppm/K) [6]. It follows that, at room temperature, the length of a body differs only slightly from its length at 0 °C. The experimental determination of the average CLTE thus reduces to measuring the length of the test specimen and its temperature; this is most commonly carried out with instruments known as dilatometers [14,15]. The measurement error of the average CLTE depends on the specimen length, the temperature range (typically ~100 °C), and the instrument type. For a number of materials (metals, glass, plastics, etc.), CLTE measurement methods are standardized [10].

**Figure 1:**
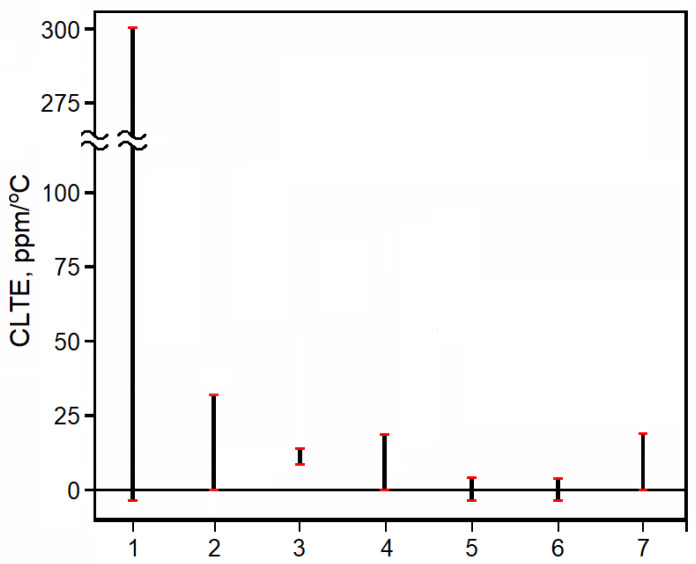
CLTE and CVTE of various materials: (1) polymeric materials at temperatures below the glass transition temperature (T_g_); (2) wood; (3) concrete; (4) metals and metal alloys; (5) inorganic crystalline materials; (6) different allotropic modifications of carbon; (7) bone tissue.

Thermal expansion coefficients depend on the chemical structure of the substance as well as on temperature. Figure 1 summarizes the orders of magnitude of the CLTE across representative classes of solids [16,17]. Polymers below T_g_ exhibit comparatively high CLTE and values span a very wide range (up to ~300 ppm/°C) because CLTE of polymers depends on macromolecular chain stiffness (greater chain flexibility corresponds to higher CLTE) and on the presence and volume fraction of crystalline phase. Wood—whose structural polymer is the rigid-chain cellulose—shows low, compared with most polymers, CLTE ≈ 0–30 ppm/°C with a broad spread due to strong anisotropy between longitudinal and transverse directions. Concrete exhibits low CLTE (8–12 ppm/°C) because its low-expansion mineral aggregates form a stiff load-bearing skeleton that constrains thermal strain in the cement paste, thereby reducing macroscopic thermal expansion of the composite. Metals and metal alloys have CLTE values between 0 and 20 ppm/°C; their CLTE is governed by the type of crystal lattice and varies with elemental composition and alloy state. Most inorganic crystalline solids and several carbon allotropes have CLTE values close to zero due to lattice-dynamical effects in stiff ionic/covalent networks. Bone tissue shows intermediate CLTE (up to ~20 ppm °C^−1^) between metals and polymers, consistent with its mineralized-collagen composite architecture and requiring careful selection of implant materials. There are also substances that have a negative CLTE; materials made from them contract upon heating, but this usually occurs only over a narrow temperature range [17]. There are also substances whose CLTE is approximately equal to zero in a certain temperature range [18,19,20,21].

The need to account for changes in the length (and volume) of solids with temperature change can be illustrated with simple examples (Table 2) [16,17].

These examples underscore the practical importance of the installation of expansion joints and gaps in building and bridge structures, gaps between rails, temperature compensators in pipelines, as well as performing temperature-dimensional calculations in mechanical engineering, engine design, electronics, optics, instrument making, and aerospace. The importance of taking into account and managing the CLTE of materials used in modern spheres also cannot be overestimated. For example, in microelectronics, the mismatch between the expansion of the substrate and the conductive layer leads to the destruction of connections between components of electrical circuits [21,22,23]. In aerospace structures subjected to temperature excursions from −180 °C to +150 °C, high CLTE values can lead to catastrophic deformation [24,25]. In precision optics, even sub-micrometer lens displacements distort the image [26]. In medical implants, a mismatch between the CLTEs of an implant and a bone can provoke rejection [27,28].

Historically, a special place among materials with very low thermal expansion is held by the metal alloy known as Invar (~64 wt.% Fe/36 wt.% Ni), discovered in 1896 by Charles Édouard Guillaume, who was awarded the Nobel Prize in Physics in 1920 for this discovery [29,30]. The unique property of this alloy—a near-zero coefficient of linear thermal expansion, 1.2 ppm/°C over 0–100 °C—arises from magnetostrictive compensation of the thermal expansion of its face-centered cubic lattice. For decades, Invar remained an indispensable material for precision instruments, length standards, components of spacecraft, and cryogenic equipment—applications in which even micrometer-scale dimensional changes under temperature fluctuations are unacceptable. However, its significant limitations—high density (~8.1 g/cm^3^), significant cost (especially for large structures), tendency to corrosion, and complexity of processing—have stimulated an intensive search for alternatives. One solution has been the development of PCMs with low CLTE that meet modern requirements for low mass (PCMs are 4–6 times less dense than metals), corrosion and chemical resistance, manufacturability of complex shapes, and reduced cost [31,32]. For this reason, polymer composites—combining the properties of the matrix (often chemically modified) with fillers having near-zero or negative CLTE (e.g., ceramics, carbon fibers)—open a pathway to a new generation of lightweight, thermally dimensionally stable, and reliable materials, including for service in extreme environments [33,34,35,36].

Despite the obvious prospect of developing PCMs with a controlled and low CLTE, and a large number of experimental studies conducted in this area, to date, virtually no scientific papers have been published that consolidate and systematize the results achieved in this area. Accordingly, the objective of this work is to provide a comprehensive review of the literature on CLTE—covering measurement methods, comparing this parameter across different types of polymer matrices, and approaches to its reduction. Special attention in the review is devoted to the consideration of methods for mathematical prediction of the CLTE of composite materials, as well as the physical and chemical principles of its regulation. The article concludes with a brief overview of application areas of polymers and PCMs in which controlling and lowering CLTE is critically important.

## 2. CLTE Measurement Methods

Accurate measurement of the CLTE of polymers and their composites is a fundamental task in the development of materials for precision and thermally loaded parts and structures, where uncontrolled deformations cause delamination, cracking, and loss of the product’s load-bearing capacity and functionality. The specific properties of polymer systems such as a low softening temperature and a pronounced decrease in the elastic (Young’s) modulus at temperatures above T_g_, the presence of viscoelastic deformations, the anisotropy of reinforced structures, and hygroscopicity, necessitate the use of specialized techniques that differ from the approaches used for metals or ceramics [37]. A particular challenge is the analysis of materials in a highly elastic state (above T_g_), where contact methods can distort the results due to specimen deformation under load [38]. For this reason, many contemporary studies combine standardized contact techniques (thermomechanical analysis, dilatometry) with non-contact optical approaches, which provide correct measurements for soft matrices. Also, the CLTE measurement devices used for polymer materials often include a vacuum-sealed or inert gas-filled chamber to eliminate the effects of hygroscopicity and thermal degradation of polymers [39,40]. In addition, for anisotropic polymers and composites, separate measurements are required along each principal axis. Thus, the choice of a CLTE measurement strategy is governed by material morphology (bulk specimens, thin films, fiber-reinforced composites), the target temperature range, and the requirements for assessing anisotropy, as detailed below in this section [41].

### 2.1. Contact Dilatometry

**Figure 2:**
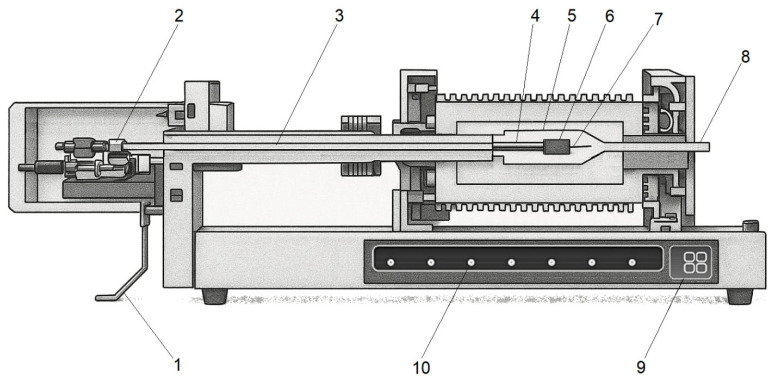
Principal scheme of a horizontal push-pull (rod) dilatometer: 1—inert-gas inlet or vacuum port; 2—Linear Variable Differential Transformer (LVDT) displacement sensor; 3—sample holder; 4—push rod; 5—furnace; 6—sample; 7—thermocouple; 8—inert gas outlet or vacuum release; 9—control panel; 10—display.

Contact dilatometry is the most widely used method for determining dimensional changes in a specimen induced by external stimuli such as heat, pressure, electric and magnetic fields, ionizing radiation, or other factors [42,43]. Figure 2 illustrates the principle of the method: minimal changes in the linear dimensions under controlled heating/cooling are recorded by mechanically transmitting the displacement via push rods to a high-precision transducer (linear variable differential transformer, LVDT). These push rods are made of materials with extremely low thermal expansion coefficients, typically quartz, Al_2_O_3_, or Invar alloy to minimize the contribution of thermal expansion of the measurement system [44,45].

When investigating polymeric materials using vertical dilatometers, the contact load should be minimized and limited to the weight of the push rod (0.01–0.1 N) to prevent deformation of soft matrices at temperatures above T_g_. In modern instruments, the influence of this factor can be reduced by holding the contact rod with a magnetic field, as well as by using piezoelectric or porous Al_2_O_3_ to reduce the mass of the rod [46]. The advantages of this method include its simplicity and accessibility, but it has lower accuracy compared to thermomechanical analysis and is not suitable for determining the CLTE of elastomers and soft rubbers.

### 2.2. Thermomechanical Analysis

The principle of the thermomechanical analysis (TMA) is to record the change in the sample’s dimensions under controlled mechanical stress during programmed heating/cooling [43]. Unlike push-rod dilatometry, where displacement is measured passively, in TMA the load (0.001–1.0 N) is applied actively, which makes it possible to simulate the real-world operational loads and to register not only thermal expansion but also softening, shrinkage, and creep [47]. A controllable load down to 10^−4^ N is achieved by varying the magnetic field acting on a ferromagnetic core coupled to the indenter, which is automatically lowered until contact with the specimen surface is detected by a touch sensor. A piezoelectric element then dynamically displaces the indenter upon detection of specimen deformation to maintain the applied load, thereby avoiding instrument-induced deformation of soft materials above T_g_ [43].

### 2.3. Optical Methods

The principle of operation of optical methods for determining the CLTE is based on the non-contact tracking of sample deformation during heating using either the analysis of the obtained images or the interaction of light waves. The key advantage of optical methods is the absence of mechanical contact with the specimen, which eliminates instrument-induced deformation n of soft polymer materials [48].

#### 2.3.1. Digital Image Correlation

**Figure 3:**
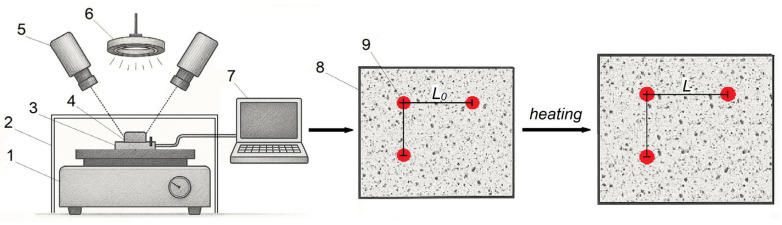
Schematic illustration of CLTE measurement by digital image correlation: 1—heating plate; 2—light transparent thermostat; 3—sample holder; 4—sample; 5—high-speed camera; 6—light source; 7—computer display; 8—sample surface digital image; 9—contrast marker; *L*_0_, *L* —the length between two markers at initial and final temperatures of measurement, respectively.

A high-speed camera (≥500 fps) records the displacement of the natural texture of the sample surface or special markers applied to it; the image sequence is then processed by the instrument software [49] (Figure 3). Using two or more cameras arranged at different viewing angles enables 3D reconstruction of surface displacements, allowing determination of CLTE both in-plane and in out-of-plane directions. The advantages of this method include the ability to simultaneously measure the thermal expansion in multiple directions, which is particularly important for an-isotropic materials. However, it requires the application of contrasting markers on the samples and has a relatively high measurement error (5–10 ppm/°C).

#### 2.3.2. Laser Interferometry

**Figure 4:**
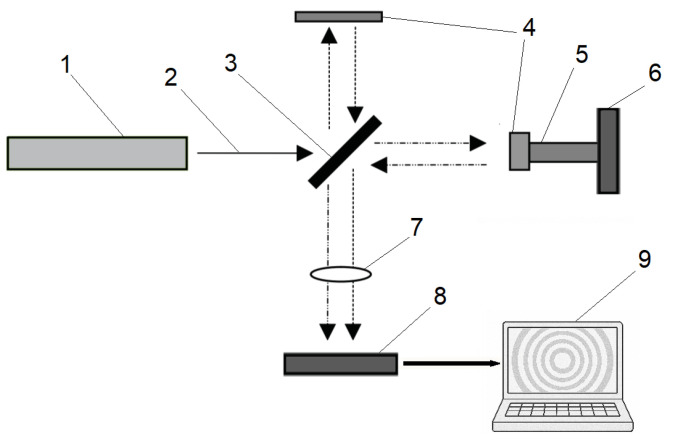
Block diagram of the experimental setup for CLTE measurement by laser interferometry method: 1—laser source; 2—laser beam; 3—beam splitter; 4—mirrors; 5—sample; 6—sample holder; 7—lens; 8—detector; 9—computer display.

A laser beam (usually a helium-neon laser with a wavelength of λ = 632.8 nm) is split into two beams: the reference beam is reflected from a reference mirror, and the measurement beam is reflected from the surface of the sample (Figure 4). The interference of the waves creates a pattern of bands, and the shift in the bands is used to determine the change in the length of the sample [50]. The method offers high accuracy, making it suitable for studying thin polymer films. Its principal drawback is the need to prepare a smooth, reflective specimen surface, typically by polishing or by depositing a metal coating (e.g., Al, Au, Pt).

#### 2.3.3. Laser Diffraction

The principle of the method is based on laser illumination of a specimen containing an optical slit (50–100 μm wide); as the temperature changes, the slit width changes, which in turn modifies the angular distribution of the diffraction-fringe intensity [51]. The advantage of this method is its high accuracy, but it requires prior specimen surface preparation (cleaning, polishing/lapping, and shaping to the required geometry).

### 2.4. Strain-Gauge Method

The method is based on the measurement of the deformation of the sample surface at a change in temperature, recorded by a strain gauge (strain resistor) [52]. When heated, the sample material expands, causing a mechanical deformation of the strain-sensitive area of the sensor, which leads to a change in its electrical resistance. The advantages of the method include the ability to measure CLTE directly on finished parts in service environments, the possibility of measuring the anisotropy by simultaneously using several gauges, and the cost-effectiveness due to the multiple reuses of strain gauges. The disadvantages of the method include the need for good adhesion of the sensor to the material, which is usually achieved by using an adhesive that has its own CLTE and forms a permanent connection with the material or product.

### 2.5. X-Ray Diffractometry

**Figure 5:**
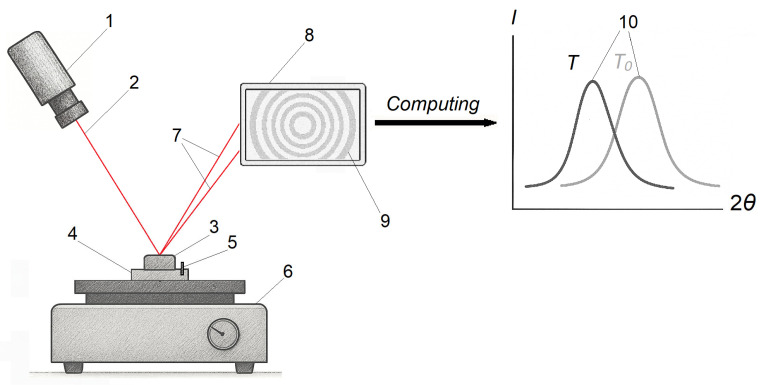
Schematic illustration of CLTE measurement by X-ray diffractometry: 1—X-ray source; 2—incident monochromatic X-ray beam; 3—sample; 4—sample holder; 5—thermocouple; 6—heating plate; 7—diffracted X-ray beams; 8—detector; 9—X-ray diffraction pattern; 10—diffraction peaks at initial and final temperatures of measurement *T*_0_ and *T*, respectively; *I*—X-ray intensity; *2θ*—the angle between the diffracted incident X-ray beams.

The method is based on the phenomenon of X-ray diffraction on the crystal lattice of the material [53,54]. When the sample is irradiated with a monochromatic X-ray beam, the atomic planes of the crystal act as reflecting surfaces, forming diffraction peaks at the diffraction angle 2θ (the angle between the incident X-ray beam and the diffracted beam). Upon heating, thermal expansion of the crystal lattice shifts the peak positions, typically to lower 2θ, which can be used to calculate the CLTE of the crystals (Figure 5). The main disadvantage of this method is that it is only applicable to crystalline polymers, while the amorphous part remains outside the scope of analysis, resulting in discrepancies of 15–40% with macroscopic measurements. However, the method offers several advantages, including atomic-scale accuracy, exceeding that of macroscopic techniques, phase selectivity, and the ability to analyze anisotropy along crystallographic axes.

Taken together, the methods reviewed above provide coverage of the practical temperature range and make it possible to determine CLTE, with sufficient accuracy, for both isotropic and anisotropic polymer and composite materials of different morphologies (bulk specimens, thin films, layered structures, fiber-reinforced structures, and particulate-filled composites). Nevertheless, no single technique provides uniform reliability for all of these material classes. The comparability of results obtained by different methods is still constrained by differences in applied load, moisture control, temperature range, surface preparation, and specimen geometry, which is especially critical for thin films and highly anisotropic composites; standardization of these test conditions remains an unresolved task. In practice, the selection of a CLTE measurement strategy should start with the material morphology and the target temperature interval, and in many cases a combined use of two or more methods is required to obtain reliable, complete data that consistently describe both the macroscopic behavior and the structural features of the material.

## 3. Mathematical Modeling Approaches for Predicting the CLTE of PCMs

The application of mathematical modeling approaches is an important method for evaluating the CLTE of PCMs. In mathematical modeling, the determination of CLTE is often reduced to the application of various effective medium theories (EMTs), which, apart from CLTE, are also employed to solve engineering analysis problems for composite structures. A number of these effective medium approaches are reviewed in [55], including the various so-called homogenization methods based on them. In this context, the problem of determining the effective physicomechanical characteristics of CMs is often referred to as the “homogenization problem” [56,57].

**Figure 6:**
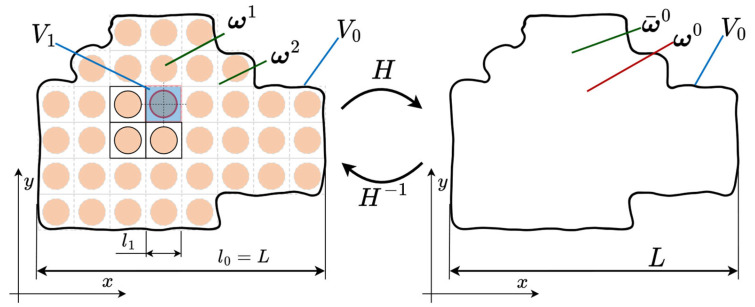
The principle of direct homogenization of the studied CM: $V_{0}$—composite domain, $V_{1}$—representative volume element (RVE), $l_{1}$—characteristic size of the RVE, $\omega^{k}$—properties (e.g., elastic moduli, CLTE, etc.) of composite component k, ${\bar{\omega }}^{0}$, $\omega^{0}$—effective composite properties obtained experimentally and numerically, respectively, H, $H^{-1}$—the operators of direct and particular case of inverse homogenization, respectively.

From a mathematical standpoint, homogenization problems can be formulated in direct and inverse forms. The direct homogenization problem consists of determining the effective physicomechanical properties of a composite (including, among others, the stiffness tensor $\bar{C}$, the thermal conductivity $\bar{\lambda }$, the thermal expansion tensor $\bar{\alpha }$, etc.) on the basis of the known properties of the constituent materials and the reinforcement architecture of the composite under study (Figure 6).

The solution of the direct homogenization problem is relevant in the context of automating the solution of a more complex and integrated task—the automated design of composites with prescribed properties—which represents the “inverse homogenization” problem.

Inverse homogenization problems can be formulated in a variety of ways. For example, if the required physicomechanical properties of the composite being designed are specified (including allowable ranges for CLTE or other parameters), the properties of the constituent materials are only partially known, and the manufacturing process is possibly defined, then, in general, the following tasks must be addressed [58,59,60,61]:

- to identify the constituent materials (in particular, the filler and the matrix), which leads to an inverse coefficient problem in the mechanics of composite materials [62];
- to identify the reinforcement scheme (particulate, fibrous, etc.) and/or the geometry of the reinforcing elements and/or the filler content (volume or mass fraction), which leads to a global structural optimization problem.

In the context of the present work, the effective tensor of thermal expansion, and, for small thermal deformations, the effective CLTE tensor $\bar{\alpha }$, constitutes the main interest. However, a detailed consideration of all existing principles for its determination and of the corresponding analytical relations lies beyond the scope of this materials science review. Therefore, we only identify several of these principles, with references to the original works, so that interested readers can consult them directly:

- Rule of mixtures [63,64,65,66,67]
- Variational approaches and analytical models [68,69,70,71,72]
- Classical laminate theory (CLT) [73,74,75]
- Asymptotic averaging (AH) [76,77,78,79]

Thus, mathematical modeling constitutes a powerful tool for predicting the CLTE, since it reduces the amount of costly and labor-intensive experimental work. In direct formulation, homogenization is used to calculate the CLTE of a composite on the basis of the known properties of its constituents (matrix and filler) and the reinforcement scheme. In the inverse formulation, homogenization is used for the targeted design of materials with prescribed properties: it enables the selection of the matrix and filler materials, as well as the optimal reinforcement scheme, shape, orientation, and volume fraction of the filler to achieve the required CLTE values. However, the highest accuracy of modeling approaches is achieved for materials with a regular or periodic microstructure, which is best suited for mathematical description and averaging. For this reason, the development of more universal methods capable of predicting various properties of composites, including CLTE, remains of considerable research interest. In this regard, recent studies have paid particular attention to computer-aided modeling techniques employing various artificial-intelligence (AI) based models [80,81,82,83,84].

## 4. CLTE of Polymer Materials

### 4.1. CLTE of Thermoplastics

**Figure 7:**
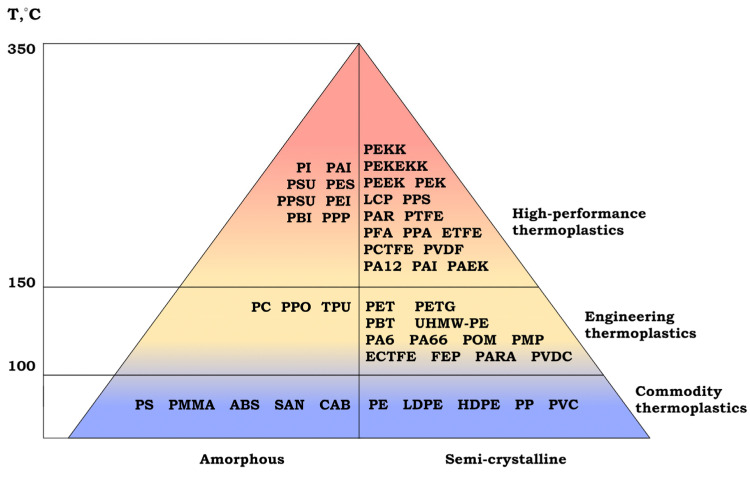
The main representatives of various classes of thermoplastics.

Thermoplastics are polymer materials that, upon heating, can reversibly transform from a glassy state to rubber elasticity and then a viscous-flow state, making them suitable for processing into products using melt methods. Thermoplastics are conventionally classified into three main groups—commodity, engineering, and high-performance—distinguished by mechanical properties, heat resistance, and applications (Figure 7) [85]. Commodity thermoplastics are characterized by relatively low cost and good processability, making them indispensable for mass production of packaging, consumer goods, and building materials [86]. Engineering thermoplastics exhibit enhanced strength, wear resistance, and temperature resistance and therefore find use in the automotive industry, electrical and electronic applications, and the manufacture of precision parts. High-performance thermoplastics combine exceptional thermal and chemical resistance, thermal stability, and mechanical strength, enabling their use in aerospace, medicine, and other fields with extreme service conditions [87,88].

**Figure 8:**
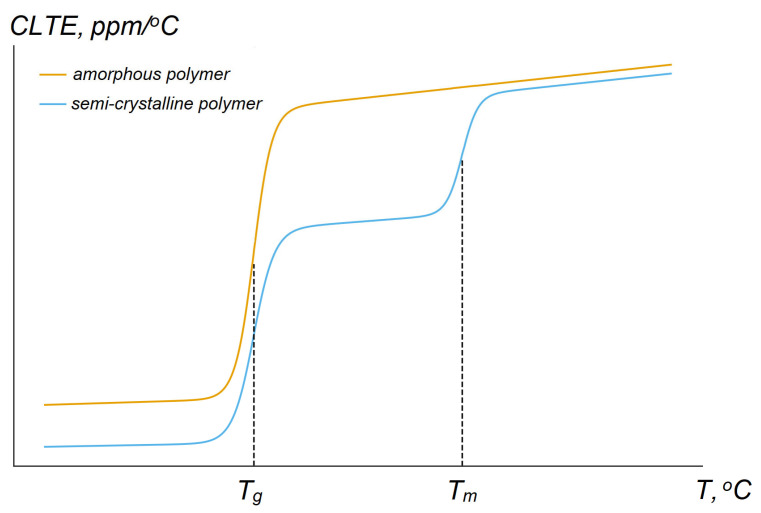
Typical temperature dependence curves of CLTE for amorphous and semi-crystalline polymers, where *T_g_* is the glass transition temperature (common to both curves); *T_m_* is the melting temperature of the semi-crystalline polymer.

The CLTE is a fundamental characteristic of thermoplastics that governs their behavior under temperature fluctuations and largely determines their suitability for practical applications [89]. In addition, this parameter is particularly important for polymer materials due to their significantly higher values compared to metals and ceramics, which requires special attention when designing products. In general, thermoplastics exhibit a broad range of CLTE values—typically 30–200 ppm/°C, and in some cases beyond this interval [34] (Table 2). This behavior is dictated by features of their molecular structure: the presence of thermal fluctuations in various groups of atoms in both the main and side chains; weak intermolecular interactions—van der Waals and hydrogen bonds, that are easily “stretched” when heated; the flexibility of macromolecules; an amorphous structure that tends to expand significantly when heated; and segmental mobility at temperatures above T_g_. For these reasons, amorphous polymers usually exhibit higher CLTE values in the glassy state than semicrystalline materials, and the transition through the glass transition temperature leads to a sharp increase in CLTE by a factor of about 2–3 or more (Table 2), which must be taken into account when parts are used over a wide temperature range [90]. This is because, below T_g_, both amorphous and semicrystalline materials are in the glassy state, in which segmental mobility is very limited, and therefore the CLTE is relatively low. Between T_g_ and T_m_, the amorphous fraction of polymers enters the rubbery (highly elastic) state and tends to expand, but crystalline lamellae (and tie chains) act as a load-bearing framework that constrains the rubbery phase; therefore, the effective CLTE of semicrystalline polymers in this region remains lower than that of amorphous polymers at the same temperature. Above Tm, the crystalline constraints are removed as the lamellae melt, so the CLTE of semicrystalline materials approaches that of the amorphous melt (typically still slightly lower due to residual crystallites and/or orientation effects). The typical dependence curve of CLTE for amorphous and semi-crystalline polymers is illustrated in Figure 8. As the degree of crystallinity increases, the polymer’s CLTE decreases, because in crystalline regions a significant fraction of the macromolecules are densely packed and coupled by intermolecular interactions of polar groups, so thermal vibrations of atoms do not produce large dimensional changes in the ordered phase. However, real materials may display more complex behavior due to crystal defects, anisotropy, and other factors.

#### 4.1.1. Commodity Thermoplastics

Commodity thermoplastics are a class of polymeric materials intended for service at relatively low temperatures (up to about 100 °C) [91]. The most common commodity thermoplastics are polyethylene (PE), polypropylene (PP), polystyrene (PS), poly(vinyl chloride) (PVC), poly(methyl methacrylate) (PMMA), and others. Members of this class exhibit large differences in CLTE, which affects their applications across various industries.

For example, PE—the most widely used thermoplastic—exhibits one of the highest CLTE values (100–300 ppm/°C), which is attributable to its flexible molecular structure and, in particular, the low degree of crystallinity (characteristic of low-density polyethylene, LDPE) [92,93]. This limits its use in precision parts, but it is compensated by its high plasticity and ability to undergo thermal deformation without failure. PP, having a more rigid molecular structure and a high degree of crystallinity, has a slightly lower CTE (80–150 ppm/°C), which, combined with its higher heat resistance, makes it preferable for products subjected to moderate thermal loads, such as food containers and interior components in residential buildings and cars [94]. Polystyrene (PS), a typical amorphous thermoplastic, exhibits a comparatively low CLTE (60–100 ppm/°C) in the glassy state, which allows its use in products that require shape stability at room temperature [95]. However, its brittleness at low temperatures and a sharp increase in thermal expansion at temperatures higher T_g_ significantly limit its temperature range of use. PVC, especially its rigid grades with a noticeable crystalline fraction (up to ~20%), shows one of the lowest CLTE values (50–80 ppm/°C) among commodity thermoplastics, which, combined with good mechanical strength, makes it widely used in construction for window profiles, piping systems, and flooring [88].

Thus, the spread in CLTE values among commodity thermoplastics is attributable to differences in their molecular architecture—the presence of bulky side groups and the nature of intermolecular interactions, the degree of crystallinity, and the flexibility of the polymer chains—which must be taken into account when designing products, especially for parts intended to operate under variable thermal loads or requiring precise dimensional compliance. The CLTE values for most commodity thermoplastics are summarized in Table 2.

#### 4.1.2. Engineering Thermoplastics

Engineering thermoplastics with heat resistance up to about 150 °C, such as poly(ethylene terephthalate) (PET), polyamides (PA), polycarbonate (PC), poly(butylene terephthalate) (PBT), and others, differ from commodity thermoplastics not only in their higher mechanical strength and heat resistance but also in a more controllable CLTE (Table 3). Their CLTE is generally lower than that of PE or PP, which makes them better suited for precision mechanical engineering, electrical/electronic applications, and other areas where dimensional stability under temperature changes is critical [96].

Particular attention should be paid to the thermal behavior of PET, whose CLTE depends significantly on the state of the polymer: in the glassy state (below ~70–80 °C) it is only 20–50 ppm/°C, approaching the values of the best engineering plastics; while in the rubbery state its CLTE rises sharply to several hundred ppm/°C [97]. This property, together with high strength and transparency, makes PET an ideal material for food packaging, e.g., blow-molded plastic bottles. In practice, preforms and heated metal blow molds are designed taking into account not only the shrinkage and expansion of PET but also for its CLTE, which makes it possible to obtain bottles with a uniform wall thickness and a predetermined shape preserved during cooling after manufacturing.

Aliphatic polyamides, depending on the type (PA6, PA66, etc.) exhibit CLTE values in the range of 70–130 ppm/°C, therefore the service temperature can significantly affect their thermal behavior [98,99]. Polycarbonate (PC), known for its impact strength and transparency, has a relatively low CLTE (≈60–70 ppm/°C), which makes it suitable for use in dimensionally precise optical systems [100]. Unlike PET, the CLTE of PBT remains comparatively stable over a wider temperature interval (≈80–90 ppm/°C up to ~160 °C), which broadens its applicability. Poly(phenylene oxide) (PPO) exhibits one of the lower CLTE values among engineering thermoplastics (≈40–60 ppm/°C), which, with its low hygroscopicity, makes it suitable for precision parts operating in humid environments [90].

Thus, a controlled and predictable CLTE is one of the key advantages of engineering thermoplastics over commodity thermoplastics. This enables their use in critical instrument and machine components where not only strength and heat resistance but also dimensional stability over a wide temperature range are required [34].

#### 4.1.3. High-Performance Thermoplastics

High-performance thermoplastics are a special class of advanced polymeric materials characterized by exceptional heat resistance and low CLTE [101]. Materials such as poly(ether ether ketone) (PEEK), polyimides (PI), polyetherimides (PEI), polyamide-imides (PAI), poly(phenylene sulfide) (PPS), and liquid-crystal polymers (LCPs) exhibit CLTE values in the range 0–70 ppm/°C, comparable to some metals and far below those of commodity and engineering thermoplastics (Table 3) [34]. For example, depending on the degree of crystallinity, PEEK shows CLTE values below 50 ppm/°C at temperatures up to about 120 °C while retaining high mechanical strength up to 250 °C, which underpins its widespread use, e.g., in aerospace [102]. Evidently, the chemical structure of high-performance thermoplastics is the key determinant of their thermal properties, including CLTE. For example, in ref. [32], it was shown that amide, ester, phenyl, and imide fragments are of the greatest interest for achieving low CLTE for a number of high-performance thermoplastics.

Among high-performance thermoplastics, polyimides (PI) exhibit some of the lowest CLTE values, which is due to the strong intermolecular interactions between their macromolecules and very high glass transition temperatures (up to ~400 °C and above) [103,104,105]. As an example, a PI obtained from 3,3′,4,4′-biphenyltetracarboxylic dianhydride and p-phenylenediamine (brand name Upilex^®^) shows a low CLTE of about 10–15 ppm/°C. However, as with all thermoplastics, the CLTE of PIs depends on such factors as molecular weight, heating regime, sample or product thickness [106] and, most importantly, on the nature of the monomers it made of. For example, study [107] demonstrated a correlation between the supramolecular structure of PI and CLTE: a denser packing of PI macromolecules leads to an increase in polymer density and mechanical modulus, while reducing CLTE. This can be achieved by using diamines with para-oriented amine groups and monomers lacking “hinge” groups. Depending on the chemical structure, the density of rigid-chain PI ranges from 1.28 to 1.54 g/cm^3^, the mechanical modulus varies from 2.7 to 11 GPa, and the CLTE changes in the range of from 2 to 63 ppm/°C. It was also shown that carrying out the imidization (cyclization) reaction under pressure significantly lowers CLTE [107]. However, despite their low CLTE, PIs have a significant drawback: an extremely high melt viscosity that precludes melt processing into products and thus substantially limits their applications.

Compared to rigid-chain PIs, polyetherimides (PEIs) that contain a lot of “hinge” type bonds—such as methylene, ether, and isopropylidene groups—exhibit significantly higher CLTE values [108]. For example, the most widely used PEI sold under the trade name Ultem^®^ has a CLTE of about 50 ppm/°C [109]. Notably, PEIs are anisotropic: study [110] showed that an injection-molded PEI specimen had different CLTE values in different directions—approximately 48 ppm/°C along the flow direction during molding and 56 and 64 ppm/°C in the transverse directions—which the authors attribute to macromolecular orientation along the flow. It was also shown that this CLTE anisotropy can be nearly eliminated by reprocessing the polymer by hot pressing, yielding CLTE values averaged to 55, 57, and 58 ppm/°C along the respective axes. Furthermore, it was demonstrated in [111] that by varying the rigidity of the diamine (a precursor in PEI synthesis), the polymer’s CLTE can be regulated from −3 to 50 ppm/°C.

Among high-performance thermoplastics, PAI and LCPs exhibit the lowest CLTE values—typically 0–40 ppm/°C, and in some cases even below zero [112,113]. At the same time, they retain their mechanical properties, thermo-oxidative stability, and hence operability up to ~250 °C and above. These properties are due to the high degree of molecular order in the crystalline phase, which makes them indispensable for components in microelectronics and precision instrumentation where thermal deformations must be minimized.

**Figure 9:**
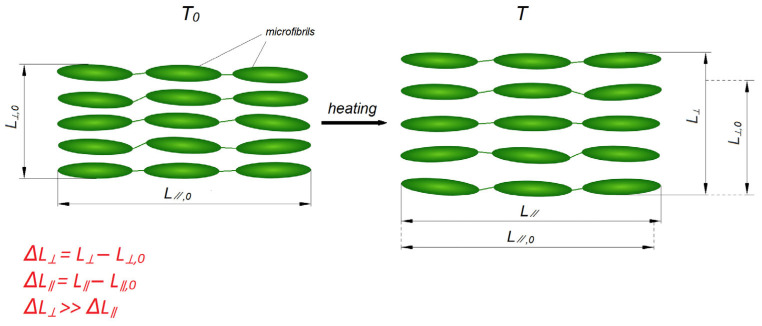
Schematic mechanism of anisotropic thermal expansion in LCPs, where *T*_0_, *T* are the initial and final temperatures, respectively; *L*_*⊥*,__0_ and *L_⊥_* are specimen dimension perpendicular to the microfibril orientation at temperatures *T*_0_ and *T*, respectively; *L*_*∥*,__0_ and *L_∥_* are specimen dimension along the orientation at temperatures *T*_0_ and *T*, respectively; Δ*L_⊥_* and Δ*L_∥_* are changes in length along and perpendicular to the microfibril orientation, respectively.

Unlike the crystal–amorphous morphology of crystallizing thermoplastics, liquid-crystal polymers (LCPs) exhibit a mesomorphic state intermediate between crystal and liquid, in which the molecules possess orientational order but do not form a crystalline lattice [114]. As a result, LCPs almost do not undergo shrinkage during processing, since they do not crystallize upon cooling. Mesophase ordering imparts anisotropy of properties, however it is not crystallization in the classical sense, since the rigid rodlike molecules of LCPs cannot fold into lamellae or spherulites as flexible-chain thermoplastics do [115]. Instead, processing induces aligned microfibrils—microfibrillar bundles of rod- or disk-like oriented units, which act as self-reinforcing structural units (Figure 9). Thus, owing to their rigid self-organized oriented structures, LCPs exhibit extremely low CLTE values along the orientation direction. For example, Vectra^®^ (Celanese)—4–12 ppm/°C, Xydar^®^ (Solvay)—5–15 ppm/°C, and Zenite^®^ (DuPont)—3–10 ppm/°C. However, transverse to the orientation direction their CLTEs are of markedly higher values (20–50 ppm/°C) [116]. LCPs outperform conventional crystallizing thermoplastics in heat resistance and mechanical properties, but their use is limited due to their high cost.

**Table 3:** CLTE of thermoplastics: α_1,_ α_2_—approximate value of CLTE of a thermoplastic in the temperature range ΔT_1_ (below T_g_) and ΔT_2_ (above T_g_), respectively.

Polymer Type	ΔT1, °C	αl, ppm/°C	ΔT2, °C	α2, ppm/°C	Source
High-density polyethylene (HDPE)	-	-	−30–60	~180	[92]
Low-density polyethylene (LDPE)	-	-	0–65	~300	[93]
Polypropylene (PP)	-	-	−50–50	~110	[94]
Polystyrene (PS)	0–100	~60–100	-	-	[95]
Poly(methyl methacrylate) (PMMA)	0–90	~150	110–150	~250–300	[117]
	20–65	~100–150			[118]
Poly(vinyl chloride) (PVC)	20–90	~80	-	-	[119]
Acrylonitrile–butadiene–styrene (ABS)	20–50	~90	-	-	[120]
Poly(ethylene terephthalate) (PET)	−40–50	~20–50	80–200	~650	[97]
Polyamide 6 (nylon 6, PA6)	0–30	~75	60–80	~130	[98]
Polyamide 66 (nylon 66, PA66)	−20–50	~70–80	100–130	~110–120	[99]
Poly(butylene terephthalate) (PBT)	−40–40	~80	70–160	~90	[121]
Poly(phenylene oxide) (PPO)	0–180	~25–75	230–260	~200	[90]
Polyphthalamide (PPA)	25–80	~80	-	-	[122]
Polycarbonate (PC)	−40–95	~60–70	-	-	[100]
Poly(phenylene sulfide) (PPS)	20–70	~50	-	-	[123]
Polysulfone (PSU)	20–80	~55			[124]
Polyethersulfone (PES)	30–150	~70			[125]
Polyphenylsulfone (PPSU)	20–80	~55	-	-	[126]
Poly(ether ketone) (PEK)	50–120	~60	-	-	[127]
Poly(ether ether ketone) (PEEK)	25–120	~45–50	180–250	~100–120	[102]
Poly(ether ketone ketone) (PEKK)	20–80	~30	-	-	[128]
Polyimides (PI)	50–250	~0–50	-	-	[107]
	50–200	~0–50			[129]
Polyetherimide (PEI)	0–150	~40–65	-	-	[130]
Polyamide–imide (PAI)	30–300	~−5–45	-	-	[112]
Liquid-crystal polymers (LCPs) –along the macromolecular orientation	20–200	~0–10	-	-	[131]
Liquid-crystal polymers (LCPs) –transverse to the macromolecular orientation	50–150	~30	-	-	[116]

### 4.2. CLTE of Thermosetting Polymers

Thermosetting polymers (thermosets) are produced via an irreversible chemical curing process (often heat-activated) that results in a crosslinked network structure. The resulting solid material is insoluble and cannot be melted, hence once the required degree of cure (hardness) is reached, the polymer cannot be reprocessed. Owing to the three-dimensional crosslinked network, thermosets generally exhibit high tensile strength, good elasticity, and the ability to operate at higher temperatures compared with thermoplastics.

Low-CLTE thermosetting plastics are in demand across many engineering fields where dimensional stability under temperature fluctuations is critical. In microelectronics and printed-circuit-board (PCB) manufacturing, thermosetting resins reduce deformation and prevent cracking upon heating, thereby improving component longevity [132]. In aerospace, where thermal gradients are extreme, they are used as matrices for structural composites due to their high mechanical properties and low density [133]. Optoelectronics and laser systems also require low-CLTE materials to avoid misalignment of optical elements and loss of accuracy [134]. In mechanical engineering—particularly for precision metrology instruments and machine tools—such resins help minimize thermally induced deformation [135]. Thermosetting resins are also employed in additive manufacturing for 3D printing of thermally stable parts; in this case, a low CLTE reduces residual stresses and improves product quality [136].

Regarding to the curing conditions, two types of thermosetting polymers are distinguished: chemically curable systems, in which the curing reaction starts upon mixing the initial components (usually two) at room or slightly elevated temperature; and thermally curable systems, for which heating is required to initiate the crosslinking reaction and to bring it to completion. The first type includes, for example, epoxy resins (ERs) cured with aliphatic amines, polyurethanes, unsaturated polyester resins cured by peroxides, and silicones. The curing reactions of these systems are generally exothermic. The second type includes, for example, resins cured on heating with aromatic amines or anhydrides, one-component phenol–formaldehyde resins (PPRs), bismaleimides (BMIs), PIs, and silicones that are cured at 120–250 °C (and at even higher temperatures, e.g., for PIs) [137].

Systems of the first type typically exhibit a relatively high CLTE range (usually 50–120∙10^−6^/°C), because the relatively low curing temperature limits the reaction rate, the macromolecular chains do not have enough time to adopt a more energetically favorable and compact conformation with low free volume, and the resulting crosslink density remains moderate; as a consequence, chain segments retain substantial mobility, which leads to higher thermal expansion [138].

Thermally cured systems exhibit a comparatively low CLTE range, approximately 10–60∙10^−6^/°C, because heating provides sufficient energy for the reaction to proceed to a higher conversion of active sites, resulting in a dense and closely spaced network with short bridges between crosslink points, which strongly restricts polymer-chain mobility and minimizes thermal expansion [139,140]. Most hot-curing systems (phenolic resins, PIs, aromatic ERs) contain rigid benzene rings that increase the Tg of the material and further reduce its thermal expansion. This explains why, in electronics, aerospace, and other high-technology fields where maximum dimensional stability under thermal cycling is required, hot-curing materials (epoxy, phenolic, and polyimide compounds) with a mandatory post-curing stage are predominant. The use of trifunctional and tetrafunctional resins or polyfunctional curing agents also contributes to obtaining materials with low CLTE. Furthermore, applying post-curing (additional heating after primary shaping) makes it possible to increase the degree of crosslinking to its maximum, reduce residual stresses, and further reduce the CLTE [141].

The most commonly used thermosetting polymers are epoxy, unsaturated polyester, phenol–formaldehyde, bismaleimide, silicone resins, and thermally cured PIs. The following sections summarize CLTE values for these materials (Table 3) and consider factors that influence this parameter.

#### 4.2.1. Epoxy Resins

Epoxy resins (ERs) are the largest thermoset class by production volume and breadth of application. They are oligomers, typically bisphenol-A (BPA) based, containing epoxide groups that are cured with crosslinking agents (aliphatic and aromatic amines, anhydrides, phenols, etc.) to form three-dimensional networks. Major application areas of ERs include aerospace composites (carbon fiber–reinforced plastics), electronic devices, adhesives, anticorrosion coatings, and construction materials. However, ERs exhibit quite high CLTE values—about 60–80 ppm/°C for BPA-based resins and 35–80 ppm/°C for modified resorcinol, novolac, and other specialty epoxies (Table 4), which limits their use in the aforementioned fields [142,143]. To reduce CLTE, epoxy matrices are often filled with particulate or fabric reinforcements having their own low or even negative CLTE, enabling composites with CLTE values down to ~0 ppm/°C. This topic is discussed in detail in Section 5.2 of the present review.

#### 4.2.2. Polyester Resins

Polyester resins are thermosetting oligomers synthesized by polycondensation of unsaturated (maleic, fumaric) and saturated (most commonly ortho-phthalic and iso-phthalic) acids or their anhydrides (e.g., maleic anhydride) with bifunctional glycols (diols) [144]. Their curing is carried out by free-radical copolymerization at the double bonds of the oligomers and, usually, of styrene, used as a reactive diluent in most commercial grades [145]. Alternative systems include polyester resins modified with epoxies and acrylic or methacrylic acid, as well as vinyl-ester and epoxy-ester resins [144]. The main applications of polyester resins are in the production of glass-fiber-reinforced plastics and polymer concretes for building panels, sanitary ware, boat hulls, swimming pools, etc. [146,147].

The CLTE and other physical properties of polyester resins are determined primarily by their chemical composition and the architecture of the three-dimensional network. However, commercial polyester resins are typically oligomers derived from several different dicarboxylic acids and glycols, which complicates systematic studies of composition–property relationships. On average, the CLTE of polyester resins is about 100 ppm/°C in the crosslinked glassy state and 180–200 ppm/°C in the rubbery elastic state (Table 4). Nevertheless, this parameter can be reduced to some extent by chemical modification of the oligomers. In ref. [148], the effect of end-group modification of an unsaturated polyester oligomer (based on maleic anhydride, ortho-phthalic acid, and various glycols) with diisocyanates of varying molecular rigidity was investigated. It was shown that introducing rigid end groups—diphenylmethane-4,4′-diisocyanate and isophorone diisocyanate—reduced the CLTE measured over 30–65 °C, from 104.2 to 81.4 and 87.8 ppm/°C, respectively. In contrast, incorporation of flexible-chain segments of hexamethylene-1,6-diisocyanate led to a slight increase in CLTE to 106.3 ppm/°C. In addition, it was shown that residual moisture in the oligomer solutions raises the CLTE of the cured polyester due to the plasticizing effect of water [149].

#### 4.2.3. Phenol–Formaldehyde Resins

Phenol–formaldehyde resins (PFRs) are synthetic thermosets produced by the polycondensation of phenol and formaldehyde in the presence of acidic or basic catalysts [150]. PFRs were first obtained in the first decade of the 20th century by the American chemist Leo Baekeland, hence PFRs are also known as Bakelite [151]. Major applications include wood-based panels, abrasive materials, mineral-wool thermal insulation, electrical insulating laminates, flame-retardant coatings, and others [152]. The key advantages of PFRs are low raw-material cost, high mechanical strength, resistance to moisture and chemicals, electrical insulating properties, and the ability to withstand short-term exposure up to about 250 °C [153]. However, due to the risk of thermal degradation under prolonged heating, the practical service temperature is limited to 100–150 °C. Significant drawbacks include brittleness, dark color of the products, the need for high molding pressures, and environmental restrictions arising from the toxicity of formaldehyde released during synthesis, processing, and use [154].

The CLTE of PFRs is on average 45–75 ppm/°C in the glassy state, which is roughly comparable to that of epoxies and markedly lower than that of unsaturated polyester resins (100–180 ppm/°C). When the temperature exceeds T_g_ (100–150 °C), the CLTE of cured PFRs rises sharply due to softening of the network, reaching about 150–200 ppm/°C (Table 4). The primary factor that reduces the CLTE of PFRs is the high crosslink density in resole resins; values as low as 40–55 ppm/°C can be achieved.

#### 4.2.4. Bismaleimide (BMI) Resins

BMIs are high-temperature thermosets synthesized from aromatic diamines and maleic anhydride. BMIs crosslink via radical polymerization at the maleimide double bonds upon heating (typically 180–250 °C) [155]. BMIs are used widely due to their very high heat resistance (T_g_ > 250 °C), flame resistance, low moisture uptake, and excellent electrical insulating properties [156]. In essence, BMIs share many of the advantages of PIs, yet they can be processed by the methods similar to those used for ERs, having much higher heat resistance. For this reason, BMIs are often used as matrix in fabric- or dispersion-reinforced composites, to produce high-performance, heat-resistant composites for aerospace (rocket and antenna fairings, fuselage and wing segments), electronics (insulators, flexible printed circuits), additive manufacturing (3D printing), and other high-technology applications [157,158]. However, due to the rigidity of the macromolecular chains and the high crosslink density after curing, BMI systems are relatively brittle, with low impact strength, fracture toughness, and elongation at break, which limits their use.

Like other thermosets, unmodified BMI resins exhibit relatively high CLTE values, on the order of 50–70 ppm/°C (Table 4). However, this parameter can be markedly reduced by modifying the BMI network structure or by incorporating inorganic fillers. For example, ref. [159] reports BMIs with near-zero and even negative CLTE achieved by using a newly synthesized diamine based on disubstituted benzocyclobutene.

#### 4.2.5. Silicone Resins

Silicone resins are a class of polymers with a backbone of alternating silicon and oxygen atoms (–Si–O–), modified with organic groups such as methyl, phenyl, and vinyl, among others [160]. Their high thermal stability (up to ~300 °C), hydrophobicity, electrical insulating properties, and optical transparency make them widely used in the industrial sector. The primary application is in coatings: electrical insulating varnishes for transformer windings, moisture-proof impregnations for concrete and brickwork, and optically transparent protective coatings for electronics that shield components from aggressive environments, mechanical damage, and UV degradation [161]. In adhesives, they are a key component of self-adhesive materials used in medical patches, mounting tapes, and electronic displays, owing to the combination of strong adhesion with easy peel. Additional uses include cosmetics (as fixative components in hair sprays imparting gloss and resilience), antifoaming agents for oil refining, and ceramic precursors in pyrolysis [162,163,164].

The CLTE of silicone resins depends on the structure of their repeat units, the degree of crosslinking, and the presence of fillers. Owing to the large number of flexible –Si–O– bonds, linear polysiloxanes with low network density—such as polydimethylsiloxane (PDMS) and poly(methylphenylsiloxane) (PMPS)—exhibit CLTE values of about 100 ppm/°C and higher (Table 4). In contrast, densely crosslinked silicone resins, for example, polymethylsilsesquioxane, have much lower CLTE values, of about 30 ppm/°C. At the same time, such resins are often characterized by anisotropic properties associated with a preferred orientation of substituent groups along one direction that can arise during curing [165].

**Table 4:** CLTE of thermosets: α_1,_ α_2_—approximate value of CLTE of a thermoset in the temperate range ΔT_1_ (below T_g_) and ΔT_2_ (above T_g_), respectively.

Polymer Type	ΔT_1_, °C	α_1_, ppm/°C	ΔT_2_, °C	α_2_, ppm/°C	Source
Epoxy resins	40–80	73	120–200	216	[166]
	50–110	65	130–210	190	[167]
	-	71	-	165	[168]
	60–100	80	200–250	183	[169]
Polyester resins	25–40	~120	70–100	~200	[170]
	20–80	~100	100–120	~120	[171]
	20–50	~55	90–140	~180	[149]
	30–65	~80–110	90–125	~180–200	[148]
Phenol-formaldehyde resins	20–90	~45–50	-	-	[172]
	-	~65–75	-	~160–180	[173]
	0–100	~50–60	-	-	[174]
Bismaleimide resins	20–80	~50	-	-	[175]
	−20–150	~70	-	-	[159]
Silicone resins	-	-	-	~165–180	[176]
	-	-	100	~125	[177]
			150	~250	
			200	~425	
			25–350	110	[178]
	25–160	~15–30			[165]
Polyimides (thermally cured)	50–250	~0–50	-	-	[107]
	50–200	~0–50			[129]

Based on Table 3, thermosetting polymers can be conditionally ranked by CLTE values, which predetermines their typical areas of use. The lowest-CLTE materials (about 0–60 ppm/°C), such as thermally cured PIs are preferred for thermally cycled or dimension-critical parts in aerospace, avionics and high-temperature electronic substrates, where mismatch of CLTE must be minimized [129]. ERs, PFRs and BMIs form a broad intermediate group: (CLTE~45–80 ppm/°C). These systems are therefore used for most structural and electrical/machinery applications, where moderate thermal expansion is acceptable but other advantages—adhesion, chemical and heat resistance, dielectric properties, compatibility with fibres—are decisive. Unsaturated polyester resins show a consistently higher CLTE (mostly 80–120 ppm/°C) and are therefore chosen for large or less dimension-critical commodity products where low material cost and simple processing are more important than tight control of thermal expansion. Most silicone resins exhibit high CLTE (≈100–180 ppm/°C and above) due to the flexible –Si–O– backbone. They are nevertheless used in electronic and optical protection because they keep their properties over a very wide temperature range, have excellent dielectric and weathering performance and are optically transparent. Furthermore, the CLTE values of silicon can be lowered by higher crosslinking degree and/or silica filling.

## 5. CLTE of Polymer Composite Materials

**Figure 10:**
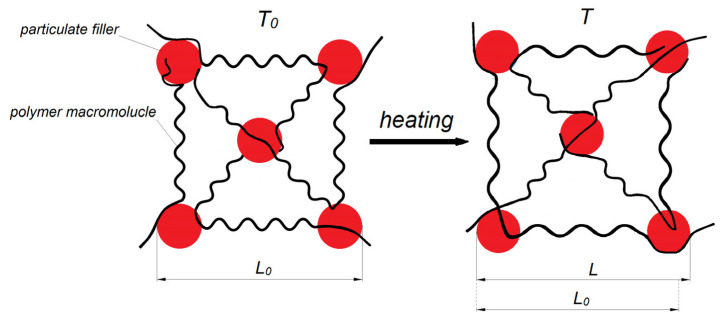
Schematic mechanism of CLTE reduction in polymer composites: filler particles mechanically constrain the polymer matrix, limiting macromolecular thermal motions upon heating; *L*_0_ and *L* are specimen dimension at initial and final temperatures *T*_0_ and *T*, respectively.

Polymer composite materials (PCMs) with controlled and, especially, low CLTE are of large practical interest for modern high-technology industries [179]. The mechanism of CLTE reduction in PCMs is that dispersed particles or continuous fibers of a filler with low CLTE and high elastic (Young’s) modulus mechanically constrain the polymer matrix, limiting thermal motions of its macromolecules upon heating [180] (Figure 10). This suppression of macromolecular mobility is particularly effective when strong interfacial adhesion is achieved at the phase boundary [181].

A mismatch in CLTE between components in microelectronic assemblies, optical systems, precision machinery, and aerospace structures under temperature gradients leads to substantial thermal stresses, deformation, delamination, and ultimately to the failure of devices [89]. For this reason, reducing the CLTE of PCMs to values comparable to those of ceramics, glass, or metals is a key objective to ensure reliability and durability under thermal cycling.

PCMs with controlled CLTE are principally divided into two main classes: particulate-filled systems, in which particles or chopped fibers are randomly oriented within the polymer matrix, and fabric-reinforced systems with an ordered microstructure. Composite fabrication methods range from standard thermoplastic processing operations such as extrusion and injection molding to specialized technologies including fabric impregnation, vacuum infusion, pultrusion, and others [182]. Hybrid approaches that combine multiple methods are also employed.

Given the very wide range of composites that exhibit near-zero [183,184,185,186,187,188] or even negative [189,190,191] CLTE, a detailed review of all types is beyond the scope of this work. Therefore, this section focuses on the fundamental factors that determine the CLTE of PCMs, such as the nature and properties of the matrix and filler; the volume fraction of the reinforcing component; the size, shape, and orientation of the filler; the magnitude of interfacial adhesion; and the influence of processing. In addition, Section 6 covers the most practically significant systems used in critical applications—including microelectronic encapsulation (enclosing fragile electronic components in a protective polymer shell) and printed circuit boards, precision optical components, and aerospace structures—where dimensional stability under temperature changes is most stringently required.

### 5.1. CLTE of Different Types of Fillers

Fillers for PCMs are divided into particulate and woven/fiber types. As a rule, particulate fillers used to lower the matrix CLTE are ceramic powders and various forms of carbon. Ceramic fillers reduce CLTE owing to their rigid covalent or ionic lattices, whereas carbon materials often exhibit negative CLTE due to the anisotropy of their crystalline structure. Woven fillers are primarily carbon, glass, and basalt fabrics. Such reinforcements form a macroscopic reinforcing framework that minimizes thermal expansion, primarily along the fiber orientation. The choice of filler type is governed by the required degree of CLTE reduction, manufacturability, cost, and compatibility with the polymer matrix.

#### 5.1.1. Ceramic Fillers

Ceramics are inorganic materials with covalent or ionic nature of bonding, high thermal stability (>1000 °C) and hardness. Among ceramic fillers for polymer matrices, the most commonly used are: oxides (SiO_2_, Al_2_O_3_, ZrO_2_); nitrides (Si_3_N_4_, AlN, BN); carbides (SiC, TiC, B_4_C); borides (TiB_2_, ZrB_2_); titanates (BaTiO_3_), zirconates (ZrW_2_O_8_); and several others [192,193].

##### Silicon-Containing Materials

Silicon (Si) is a semiconductor with a moderate CLTE of about 2.5–3 ppm/°C at room temperature and is used, for example, in microelectronics to match the thermal expansion of substrates [194]. Quartz glass (SiO_2_) has an ultra-low CLTE of ~0.55 ppm/°C, transparency from the UV to the IR, and thermal stability up to ~1400 °C. It is employed, among other applications, in optics and viewport (porthole) windows [192]. Crystalline quartz (α-SiO_2_) exhibits anisotropic thermal expansion due to the asymmetry of Si–O–Si vibrations in the lattice: along the c-axis (the principal axis in the hexagonal/trigonal setting) its CLTE is −6.5 ppm/°C, whereas perpendicular to the c-axis it is 14.3 ppm/°C [195].

##### Aluminosilicates

Aluminosilicates are a class of minerals and synthetic materials in which aluminum partially substitutes for silicon at the tetrahedral sites of silicate crystal lattice. Their general composition can be written as xAl_2_O_3_·ySiO_2_ (for simple aluminosilicates) or xM_2_O_n_·yAl_2_O_3_·zSiO_2_ (for more complex systems), where M is a metal cation (e.g., Li^+^, Mg^2+^, Ca^2+^, etc.). Aluminosilicates exhibit low—and often negative—CLTE values due to the rigidity of the tetrahedral frameworks in their crystal lattices (Table 5).

##### Nitrides

The low CLTE of nitrides is attributable to their rigid crystal structures with a predominance of strong covalent bonds, which limit the amplitude of atomic thermal vibrations [196]. They combine high energy of covalent bonds, short interatomic distances, and symmetric crystal lattices that suppress anisotropic expansion. Boron nitride (BN) has a layered lattice with strong B–N bonds that provide high thermal stability and low expansion along the layers [197]. For aluminum nitride (AlN), a low CLTE of about 4.5 ppm/°C is associated with the dense packing of [AlN_4_] tetrahedra, which resists thermal deformation. In addition, complex nitrides containing several (up to five or six) different metals are often used as fillers in PCMs [198].

##### Other Inorganic Salts

Tungstates, molybdates, titanates, and zirconates can also be classified as inorganic compounds with extremely low (and sometimes negative) CLTE values (Table 5). These substances share rigid crystal lattices with strong interatomic bonds (W–O, Mo–O, Ti–O, Zr–O) that limit the amplitude of thermal vibrations. Their key advantage is the ability to vary properties via chemical composition: cation substitution in these salts enables regulation of the CLTE to meet PCM design requirements. Notably, some studies target materials with very large negative CLTE; a record example is Bi_0.95_La_0.05_NiO_3_ with CLTE = −137 ppm/°C [197,198].

#### 5.1.2. Carbon Particulate Fillers

All allotropes of carbon exhibit relatively low, and often negative, CLTE values. For amorphous carbon, the CLTE is about 0–7 ppm/°C [199]. The crystalline forms of carbon—graphite, graphene, carbon nanotubes, fullerenes, and others—are of even greater interest.

Graphite exhibits a low (and in the case of ideal single crystals, a negative) CLTE [200]. This is due to its strong covalent hexagonal layered structure within the basal planes, which restricts atomic thermal motion upon heating [201]. On average, the in-plane CLTE is about −1.5 ppm/°C, whereas along the direction perpendicular to the layers (the c-axis) it remains relatively high, around 25 ppm/°C [202]. In polycrystalline forms of graphite that are typical of practical fillers, the bulk-averaged CLTE is usually positive but extremely low (1–5 ppm/°C) [203].

Graphene—a two-dimensional allotrope of carbon consisting of a single-atom-thick layer—was first isolated about two decades ago by the British physicist of Russian origin K.S. Novoselov [204]. Owing to its valuable properties, it is a highly promising material for next-generation electronic devices. The in-plane CLTE of graphene is negative, ranging from about −12 to −2.5 ppm/°C over 200–400 K [205]; at room temperature it is approximately −8 ppm/°C. An out-of-plane CLTE is, evidently, not defined because its thickness is that of a single carbon atom. Graphene also exhibits extremely high electrical and thermal conductivity [206] and exceptional mechanical strength [207].

Carbon nanotubes (CNTs) are cylindrical structures formed by rolling graphene sheets; depending on the number of concentric shells they are classified as single-walled (SWCNTs) or multi-walled (MWCNTs) [208]. CNTs exhibit exceptionally high axial thermal conductivity (up to several thousand W·m^−1^·K^−1^), making them promising material for thermally conductive composites and nanoelectronics [209,210]. CNTs have a negative axial CLTE [211]. This is commonly attributed to the fact that, due to strong C–C bonding, thermal vibrations preferentially produce radial rather than axial expansion. In the radial direction, where only weak van der Waals interactions prevail, the CLTE is positive (~30 ppm/°C) [212]. Consistent with this, the radial CLTE increases with increasing inner diameter of the CNTs [213].

#### 5.1.3. Fiber and Fabric Reinforcements

**Figure 11:**
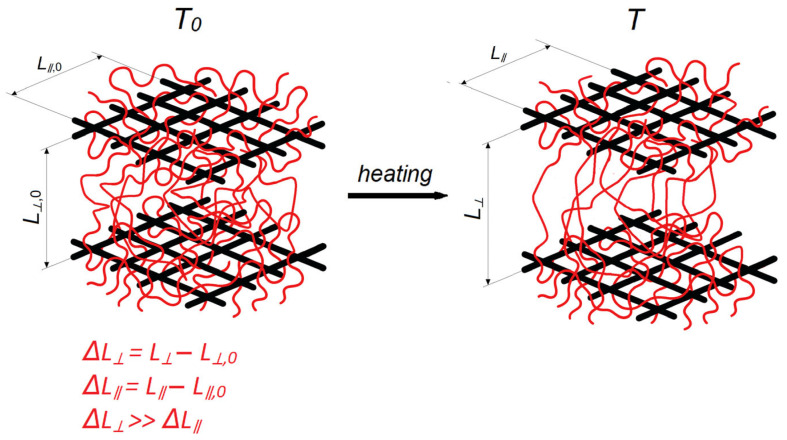
Schematic mechanism of CLTE reduction in fabric-reinforced polymer composites: woven fabric layers mechanically constrain the polymer matrix, hindering the in-plane unfolding of macromolecular chains upon heating; where *T*_0_, *T* are the initial and final temperatures, respectively; *L*_*⊥*__,__0_ and *L_⊥_* are specimen dimensions out-of-plane (perpendicular to the fabric plane) at temperatures *T*_0_ and *T*, respectively; *L*_*∥*__,__0_ and *L_∥_* are specimen dimensions in-plane (within the fabric plane) at temperatures *T*_0_ and *T*, respectively; *ΔL_⊥_* and *ΔL_∥_* are changes in specimen length out-of-plane and in-plane, respectively.

Fibrous materials—such as glass fibers and glass fabrics, carbon fibers and carbon fabrics, and basalt fibers and fabrics based on it—exhibit pronounced CLTE anisotropy between the fiber axis (longitudinal direction) and at the transverse direction, which is due to the internal molecular/crystalline structure of the filler and high degree of orientation imparted during fiber production [214]. As schematically illustrated in Figure 11, stacked woven plies mechanically constrain the polymer matrix and suppress in-plane unfolding of macromolecular chains during heating; as a result, the in-plane extension is small and the in-plane CLTE of the composite is markedly reduced compared with the neat matrix.

Glass fiber (the reinforcement used in glass fabrics). In the longitudinal direction, glass fiber exhibits a relatively low positive CLTE, typically 4.9–6.0 ppm/°C. This is attributed to the prevalence of strong covalent bonds in the amorphous silicate-glass network oriented along the drawing direction. In the transverse direction, however, the CLTE is much higher, about 20–25 ppm/°C. This anisotropy arises from the fact that transverse to the fiber, thermal expansion is determined by the behavior of less ordered regions and glass components that are not as rigidly bound in this direction. In composites, the high transverse CLTE of the fiber is partly offset by the even higher CLTE of the polymer matrix [215].

Carbon fiber (the reinforcement used in carbon fabrics) exhibits a stronger CLTE anisotropy, directly linked to its graphite-like crystalline structure. In the longitudinal direction, the CLTE is very low and often negative—typically −1.5 to +1.0 ppm/°C, depending on fiber type [216]. The higher the degree of orientation of the graphite-like planes along the fiber axis and the higher the modulus of elasticity, the more negative the CLTE becomes. In the transverse direction, the CLTE of carbon fiber is much higher—usually 7–15 ppm/°C and, for some fiber grades, up to 20–30 ppm/°C [217].

Basalt fiber has thermal properties intermediate between those of glass and carbon fibers. In the longitudinal direction, its CLTE is typically 5.0–8.0 ppm/°C. In the transverse direction, the CLTE is higher than the longitudinal value but lower than that of glass, usually 7.0–12.0 ppm/°C [218].

**Table 5:** CLTE of fillers typical for polymer composites: α—average CLTE of the filler in the studied temperature interval ΔT.

Filler	ΔT, °C	α, ppm/°C	Source
Si	25–1227	2.6–4.6	[194]
SiO_2_ (quartz glass)	25–250	0.55	[192]
Al_2_O_3_ (corundum)	25–250	6.6	[192]
	25–1000	9.1	[219]
2MgO-2Al_2_O_3_-5SiO_2_	100–600	2.0–2.8	[220]
Li_2_O-A1_2_O_3_-SiO_2_	27	−2.7	[221]
LiAlSiO_4_ (β-eucryptite), along the crystallographic a-axis.	20–1300	7.8	[221]
LiAlSiO_4_ (β-eucryptite), along the crystallographic c-axis.		−17.5	
ZnO	-	0.7	[195]
AlN	25–250	4.4	[197]
BN	-	<1	[195]
Si_3_N_4_	-	3.2	[195]
Sc_2_(WO_4_)_3_	−263–177	−2.2	[222]
PbTiO_3_	27	−4	[223]
CaTiO_3_	25–1000	13.4	[214]
BaZrO_3_	25–1000	6.3	[196]
ZrW2O_8_	−273–77	−9.1	[224]
Zr_2_WP_2_O_12_	25–800	−3.9–(−2.6)	[225]
(Mn_0.96_Fe_0.04_)_3_(Zn_0.5_Ge_0.5_)N	43–113	−25	[197]
Bi_0.95_La_0.05_NiO_3_	27–97	−137	[198]
Amorphous carbon	20–1400	~0.5–6	[199]
Graphite (in-plane)	0–300	−0.5–(−1.5)	[202]
Graphite (out-of-plane)	0–300	20–30	[202]
Diamond powder	-	0.8	[195]
Graphene	−73–127	~−12–(−2.5)	[205]
MWCNT (axial direction)	−5–85	~−20–0	[213]
	30–60	~−12	[211]
MWCNT (radial direction)	−263–47	~30	[212]
Carbon fiber (longitudinal direction)	−200–1000	−1.5–1	[216,217,226]
Carbon fiber (transverse direction)		5.5–30	
Glass fiber (longitudinal direction)	−50–350	5–6.0	[216]
Glass fiber (transverse direction)		22–25	
Basalt fiber (longitudinal direction)	−50–600	5.0–8.0	[218]
Basalt fiber (transverse direction)		7.0–12.0	

### 5.2. Key Factors Governing the CLTE of Composite Materials

#### 5.2.1. Influence of Polymer Matrix Properties

As shown in Section 4 of this review, the intrinsic CLTE of the polymer matrix is determined by the molecular mobility of the chains, the packing density of the macromolecules, and the degree of crystallinity, which are determined by the chemical structure and flexibility of the polymer chain. Rigid aromatic motifs (e.g., phenyl rings, biphenyl units) and a high crosslink density restrict conformational changes upon heating, thereby lowering the CLTE, whereas flexible aliphatic segments increase the amplitude of thermal motions and thus the thermal expansion [103,227,228]. For example, in ref. [229] the CLTE of chopped–carbon-fiber polymer composites were tuned to 30–40 ppm/°C by varying the chemical nature of the thermoplastic matrix. Moreover, many polymer matrices—typically semicrystalline ones—and the composites based on them exhibit pronounced CLTE anisotropy [110].

#### 5.2.2. Influence of Filler Properties

The nature of the filler directly determines its ability to restrict the thermal expansion of the polymer matrix. The dominant factors in this regard are the intrinsic CLTE, shape, size, orientation, surface modification, and other parameters of the filler [229]. For example, in ref. [230], epoxy-based composites containing 5 vol.% of ten different ceramic fillers exhibited CLTE values ranging from 50 to 75 ppm/°C, depending on the filler used.

##### CLTE of the Filler

Obviously, the lower the CLTE of the filler, the more effectively it suppresses polymer-chain mobility upon heating by mechanical constraint. For instance, adding 50 vol.% of three different ceramic fillers—alumina, unfunctionalized silica, and silica coated with aluminum nitride—with CLTE values of 6.6, 0.5, and 4.4 ppm/°C, respectively, reduced the composite CLTE from 88 to 38, 30, and 36 ppm/°C, respectively [192].

##### Particle Size (Dispersion) of the Filler

Another parameter that determines the CLTE of polymer composites is the particle size (dispersion) of the filler [231]. For example, study [232] examined the effect of silicon oxide particles of different dispersions (particles from 20 nm to 10 μm) on the CLTE of an epoxy composite. It was found that increasing the filler dispersion leads to a decrease in the CLTE, but the effect did not exceed ± 5 ppm/°C. In another study [233], varying the silica particle size from 0.35 to 1 μm did not lead to a systematic change in CLTE—likely because the size difference was too small to outweigh other factors. Several other publications report a modest decrease in CLTE under similar conditions. In addition, some studies have shown that reducing the length of carbon fibers used to reinforce the matrix decreases the CLTE of epoxy composites [234,235].

##### Filler Orientation

Anisotropic fillers—primarily fibrous and fabric reinforcements—sharply reduce the composite CLTE along the alignment direction (or within the alignment plane) while exerting only a limited effect in the perpendicular directions. For example, in [236] an epoxy matrix composite with aligned chopped carbon fibers exhibited a CLTE of −3 ppm/°C along the fiber-alignment axis, whereas the CLTE values were 40 and 90 ppm/°C in the in-plane transverse and through-thickness directions, respectively. Noteworthy as well are studies dedicated to deliberate orientation of particulate fillers to obtain anisotropic properties. In ref. [237], orienting iron-oxide–coated boron nitride microplatelets reduced the composite CLTE to 28.7 ppm/°C, compared with 60 ppm/°C for randomly oriented platelets.

##### Functionalization and Surface Modification of the Filler

Surface functionalization of fillers (e.g., silanization, oxidation, introduction of reactive groups) enhances interfacial adhesion—via van der Waals interactions or formation of new covalent bonds—thereby constraining the amplitude of thermal motions of polymer macromolecules upon heating and preventing the formation of microdefects during thermal cycling and service under varying temperatures. In ref. [167], thermal pre-treatment of carbon nanotubes reduced the CLTE of the corresponding epoxy composite from 60 to 40 ppm/°C. In another study, functionalizing boron nitride nanotubes with oligomeric silsesquioxane, as well as with amine or hydroxyl groups, yielded epoxy composites with CLTE values of 53, 57, and 66 ppm/°C, respectively [238].

#### 5.2.3. Composite Structure

##### Filler Content

Because the degree to which rigid inclusions occupy the matrix volume governs the mechanical constraint on thermal expansion, the filler content is one of the dominant parameters controlling the CLTE of a composite. In research practice, the filler volume fraction is used preferentially, as it is directly related to microstructure and micromechanical models; the mass fraction is employed as an auxiliary measure when accurate component densities are unavailable (e.g., porous or hybrid fillers). When the CLTE of the filler is lower than that of the matrix, the composite CLTE generally decreases with increasing volume fraction; however, the dependence may exhibit extrema or saturation due to microstructural effects, in particular the formation of a continuous (percolating) filler network within the matrix [239,240,241].

##### Interfacial Adhesion Between Matrix and Filler

The quality of adhesion at the phase interface directly governs the efficiency of transferring thermal stresses from the polymer matrix to the filler: strong interfacial coupling prevents microdefect formation (cracks, voids) during thermal cycling and ensures uniform constraint of thermal expansion. Weak adhesion leads to partial interfacial debonding, local increases in CLTE in defective regions, and reduced predictability of composite properties. Optimizing interfacial bonding (chemical modification, sizing) is therefore critical to realizing the filler’s theoretical potential for lowering CLTE.

In ref. [242], for a composite consisting of a bisphenol-A epoxy matrix and 85 wt.% silica, chemical modification of the epoxy with propyltriethoxysilane reduced the composite CLTE from 40 to 6.0 ppm/°C. Using the nuclear magnetic resonance method, it was indicated that this reduction stemmed from covalent bonds formed between the silica filler and the Si-containing substituents in the epoxy matrix, which increased interfacial adhesion and thereby lowered CLTE. Notably, the CLTE of the unmodified cured epoxy resin slightly exceeded that measured for the modified epoxy matrix, which was attributed to the plasticizing effect of the introduced propyltriethoxysilane groups. Similar trends have been reported using lower filler loadings [243] and with a different epoxy system [169]. Furthermore, as noted in the previous section, interfacial adhesion can also be enhanced by functionalizing the filler [167].

##### Hybrid Composites

Hybrid systems that combine fabric reinforcements (carbon, glass, aramid) with particulate fillers provide a synergistic reduction in CLTE through a hierarchical architecture: the fabric forms the primary stiff framework that suppresses macroscopic expansion, while nanoparticles immobilize the polymer in inter-fiber regions, minimizing microdeformations [244,245]. For example, in [246] introducing a hybrid filler consisting of glass fiber together with several mineral fillers into an epoxy matrix reduced the CLTE to 20 ppm/°C, compared with 24 ppm/°C when using glass fiber alone. In ref. [189], employing a hybrid filler of carbon fiber and zirconium tungstate yielded a negative CLTE of −2.7 ppm/°C.

#### 5.2.4. Processing Factors and CLTE Measurement Methodology

Processing and fabrication conditions of PCMs directly affect their CLTE. By varying the degree of cure (and thus crosslink density) in thermosets, the CLTE can be adjusted by about 15–30% owing to densification of the network, which restricts chain mobility [247,248]. Matrix wet-out and interfacial coverage between the matrix and the filler determine the effective interfacial contact: poor wetting leads to micropores and air inclusions that, upon heating, expand more freely than the polymer, thereby increasing the macroscopic CLTE of the composite [249,250]. Processing methods also play a key role. Injection molding and extrusion induce orientation of polymer chains and/or the filler, leading to CLTE anisotropy [110]. For thin films (<10 μm), the CLTE of polymers and their composites can differ significantly from that of bulk specimens because strong substrate–film interactions (e.g., silicon or glass) suppress in-plane expansion, reducing the in-plane CLTE by ~20–40% compared with the free-standing state [251]. At the same time, in out-plane direction CLTE of thin films may increase due to excess free volume at the polymer surface [252].

Measurement methodology is also of great importance. XRD measures the CLTE of the crystal lattice (a local expansion), neglecting the contribution of amorphous regions and thus underestimating the macroscopic CLTE by about 20–50% [53]. TMA and DMA measure the bulk CLTE but require corrections for anisotropic materials [253]. Accordingly, for composites and thin films, laser interferometry or strain-gauge techniques that directly record in-plane deformation with minimal artifacts are preferable [50,52]. Study [254] demonstrates that the CLTE of polymers depends significantly not only on the temperature range but also on the heating/cooling rate, with the discrepancy increasing as the temperature change rate rise.

Taken together, the studies analyzed in this review indicate that the use of fillers and reinforcements substantially restrict the mobility of macromolecular segments in the polymer matrix and, as a consequence, to reduce the overall CLTE of the composite down to near-zero and, in some cases, even to negative values. However, although numerous formulations with near-zero or even negative CLTE have been reported, transfer of these results to industrial components is often limited by incomplete data on the interfacial adhesion of the components, processing-induced porosity and fiber orientation, moisture conditioning, and other factors governing CLTE and other service-related properties. For these reasons, future studies should accompany CLTE measurements with a consolidated characterization of the composite microstructure (interface quality, void content, orientation and lay-up symmetry) and with tests under service-relevant conditions; this would make published data sets mutually comparable and would allow the reasons for unsuccessful attempts to achieve low CLTE to be identified explicitly.

## 6. Discussion: Applications and Practical Significance of CLTE for Polymers and PCMs

The CLTE of polymers and PCMs is critical in the design of parts and products intended to operate under temperature gradients. Accordingly, CLTE reference data constitute one of the principal input parameters can be used either to select polymer matrices or PCMs whose CLTE falls within the range of conventional materials applied in the product, or to decide on the introduction of compensating solutions (e.g., compliant interlayers, fillers, local reinforcing layers, jointing or transition elements, etc.) to mitigate thermal-expansion mismatches. Table 6 summarizes and compares CLTE values for polymers and PCMs with those of conventional engineering materials that are most frequently used under thermal loading conditions.

Polymers with low CLTE (e.g., PIs, PEEK, LCPs, PTFE, and some thermosets) are used in precision mechanics, microelectronics, and aerospace applications [255,256]. Polymers with high CLTE (PE, PP, PVC, etc.) require compensation of thermal deformations by using flexible joints, for example, in hot- and cold-water piping systems [257,258]. In PCMs, components with similar CLTE are selected to avoid delamination [259]. When combining polymer and metal parts—e.g., in printed circuit boards and instrument housings—the CLTE mismatch must be accounted for and expansion clearances (allowances) introduced to prevent failure upon heating [260].

In molding processes (injection molding, casting into silicone molds, etc.), accounting for CLTE is critical for ensuring dimensional accuracy, minimizing deformations, and pre-venting defects due to warping and cracking during heating/cooling of products and parts [261]. As it is known, polymers contract on cooling from the melt to room temperature, resulting in shrinkage (0.2–2% depending on the material). Nonuniform shrinkage—arising from flow-induced anisotropy of CLTE—produces warpage. Rapid cooling of the surface of the product while maintaining a hot core creates thermoelastic stresses that can lead to cracking. If CLTE is not considered when designing the mold, the finished products and parts may deviate from the design drawing, which is especially critical for precision components (gears, bearings, electronic parts, etc.). To avoid this, cavity dimensions are calculated via computer-aided engineering simulations that predict shrinkage and warpage while accounting for CLTE anisotropy and nonuniform cooling. The use of metallic inserts in polymer moldings leads to significant internal stresses due to the large mismatch between the CLTEs of steel and polymers, which can cause failure. Recommended mitigations include minimizing the wall thickness of the metallic insert/reinforcement and selecting a polymer with a low CLTE. Technological optimization is also important: slower cooling reduces temperature gradients and residual stresses; maintaining an optimal, stable mold temperature decreases shrinkage nonuniformity; and applying adequate packing/holding pressure compensates for shrinkage solidification (or cure, for thermosets).

The CLTE plays a crucial role in the design and fabrication of polymer components for space applications [262]. The wide operating temperature range (from −180 to +150 °C), thermal cycling, accelerated outgassing in vacuum, ionizing radiation, and exposure to atomic oxygen all alter polymer molecular weight and structure, which in turn affects CLTE. Consequently, all polymer parts and devices intended for space service—housings/cases, fasteners, adhesives, seals, electrical and thermal insulation, precision antenna elements, elements of solar batteries, etc.—must be made from materials with low CLTE and high resistance to the near-Earth and deep-space environments. For example, antenna reflectors are commonly fabricated from carbon-fiber-reinforced composites with epoxy, PEEK, or LCP matrices, while interface seals for the International Space Station modules employ modified PTFE filled with ceramic additives. Accounting for the CLTE in the design and manufacture of polymer fasteners (bolts, nuts, washers, rivets, etc.) is critical to ensure durability and functional performance under thermal-cycling conditions [263].

The use of polymers and polymer composites in cryogenic applications is associated with several key challenges: the development of internal stresses due to material contraction, the loss of tightness in elastomer seals/gaskets, a two- to threefold increase in tensile strength, and a ten- to hundredfold decrease in impact toughness. To overcome these problems, materials with very low CLTE—PIs, fluoropolymers (e.g., PTFE), PEEK, and LCPs—are employed, as well as filled epoxy resins, metal–polymer particulate composites (e.g., copper–PTFE) and multilayer composites (e.g., PI–aluminum) [264]. Properly accounting for CLTE enables the design of polymer components that remain functional even at cryogenic temperatures approaching absolute zero (−273 °C).

Accounting for the CLTE of polymer PCB substrates is critical to ensuring the reliability and service life of electronic devices, especially under thermal cycling or high-temperature operation [132]. This is because heating/cooling induces mechanical stresses due to CTE mismatch between the polymer substrate and metallic conductors (copper, aluminum), leading to trace cracking of copper traces, peeling of the conductors, breaks in the interlayer connections in multilayer boards, layer-to-layer misregistration, and problems with component attachment. Accordingly, PCB substrates—including flexible circuits—are made from low-CLTE materials such as glass-fabric–reinforced epoxies, polyethylene naphthalate (PEN), and PIs (Kapton^®^, Upilex^®^). In addition, technological methods are employed to improve reliability, for example, graded-CTE transition zones in rigid-flex boards.

In medicine, polymer implants—e.g., PEEK-Optima^®^ orthopedic prostheses and PEKK dental crowns—must have a CLTE compatible with that of bone (~10–15 ppm/°C) to prevent interfacial micromotion [265]. Fasteners (such as surgical clamps made from PPSU and bolts made from the LCP Zenite^®^) are designed with CLTE taken into account to maintain clamping force during steam-autoclave sterilization cycles.

Fiber-reinforced PCMs with a substantial volume fraction of carbon fibers can be used to fabricate so-called scale bars—thermally stable rods with low CLTE whose length is predetermined with high accuracy. Special targets are placed at the opposite ends of the bar, and the center-to-center distance is then measured, for example, with a coordinate-measuring machine. Owing to their thermal stability, such scale bars can be used over a wide temperature range in industrial photogrammetry and 3D scanning to set the absolute measurement scale, enabling a variety of industrial 3D-metrology tasks: reverse engineering, alignment and assembly of large structures, geometric inspection of manufactured parts, measurement of large objects and creation of their digital models, calibration of robotic and measuring systems, etc. [266,267]. The traditional material for scale bars is Invar alloy, which has a near-zero CLTE [29]. However, Invar is characterized by high cost, significant mass, and difficulty to produce. In recent years pultruded unidirectional carbon-fiber–reinforced PCMs have come into widespread use for this purpose [268,269]. As noted earlier, such composites exhibit extremely low CLTE in the longitudinal (fiber) direction.

Thus, the proper use of CLTE data helps prevent structural failures, extends equipment service life, preserves the accuracy of measuring instruments, and ensures the safe operation of engineering systems.

## 7. Conclusions

The present review summarizes current knowledge on the coefficient of linear thermal expansion (CLTE) of polymers and polymer composite materials (PCMs) in terms of measurement approaches, structure–property relations, and routes to CLTE reduction. It is shown that, unlike other structural materials (e.g., metals and ceramics), polymers and PCMs require specialized CLTE measurement techniques and careful control of test conditions (temperature range, moisture content, assessment of anisotropy, and other influencing factors), since these parameters strongly affect the obtained values. Furthermore, an important area of development is the further advancement of mathematical modeling approaches for predicting CLTE, which makes it possible to reduce the cost and duration of studies as compared with exclusively experimental determination.

For neat polymers, CLTE is governed mainly by the phase state (glassy or rubbery), degree of crystallinity, chain rigidity, and—for thermosets—curing temperature and crosslink density; transition through Tg typically causes a stepwise increase in CLTE by a factor of 2–3. Thermoplastics therefore cover a wide range, from 50 to 300 ppm/°C for commodity and engineering grades down to 0–10 ppm/°C for some high-performance thermoplastics such as PI, LCPs. A substantial and reproducible decrease in CLTE is achieved by forming polymer composites in which the matrix is mechanically constrained by fillers or reinforcements with intrinsically low (or negative) thermal expansion and high stiffness. The resulting CLTE is determined not only by the properties and volume fraction of the filler, but also by its orientation, interfacial adhesion, processing-induced porosity, and the symmetry of the reinforcement architecture; incomplete reporting of these parameters is the main reason for difficulties in transferring laboratory formulations to engineering structures.

Promising directions for further research, in our opinion, include: CLTE measurements under standardized and service-relevant conditions (including thermal and hygrothermal cycling); the systematic characterization of composite microstructure alongside CLTE; and the development of analytical and multiscale modeling tools (including AI-based) capable of predicting the thermal expansion of anisotropic, heterogeneous PCMs. Furthermore, research should address the targeted design of polymer matrices with controlled CLTE, clarify the role of interfacial adhesion and nanoscale effects in CLTE reduction, and explore hybrid composite architectures. Taken together, these efforts will make published data sets mutually comparable and will provide reliable design guidelines for dimensionally stable polymer-based components, including those intended for extreme operating environments.

## Figures and Tables

**Table 1:** Matrix form of the components of the thermal expansion tensor.

Anisotropy	Orthotropy (e.g., Fiber-Reinforced PCMs with a Regular Microstructure)	Isotropy (Homogeneous Materials, e.g., Amorphous Polymers)
$\left[\begin{array}{ccc} \alpha_{11} & \alpha_{12} & \alpha_{13}\\ \cdot & \alpha_{22} & \alpha_{23}\\ \cdot & \cdot & \alpha_{33} \end{array}\right]$	$\left[\begin{array}{ccc} \alpha_{11} & 0 & 0\\ 0 & \alpha_{22} & 0\\ 0 & 0 & \alpha_{33} \end{array}\right]$	$\left[\begin{array}{ccc} \alpha & 0 & 0\\ 0 & \alpha & 0\\ 0 & 0 & \alpha \end{array}\right]$

**Table 2:** Elongation of 1 m long samples made of different materials with a temperature change of 100 °C.

Material	Elongation, mm
glass	0.9
concrete	1.2
copper, stainless steel	1.7
aluminum	2.4
polypropylene	15
polyethylene	20

**Table 6:** Comparison of CLTE ranges for polymers, PCMs, and most commonly used structural and functional materials.

CLTE Range (ppm/^°C^)	Materials
**0–10**	**Non-polymeric materials:** quarts glass; borosilicate glass; ceramics (e.g., SiC, Al_2_O_3_, dental/technical glass-ceramics); special metal alloys (e.g., Invar, Kovar), wood (longitudinal direction); concrete (up to 12–13 ppm/^°C^) **Polymers:** LCPs (along the macromolecular orientation) **PCMs:** carbon fiber filled ERs, highly silica-filled PCMs
**10–20**	**Non-polymeric materials:** cement; bone tissues; gypsum; engineered stones **Polymers:** high crosslink density PI; aromatic PAI **PCMs:** glass fiber filled ERs; mineral-filled PCMs
**20–100**	**Non-polymeric materials:** wood (transverse direction); plywood; hardboard; asphalt **Polymers:** LCPs (transverse to the macromolecular orientation); PS; PVC; ABS; PET (below T_g_); PA6; PA66; PBT; PPO; PPA; PC; PPS; PSU; PES; PPSU; PEK; PEEK; PEKK; PEI; ERs; PFRs; BMIs; polyester resins **PCMs:** particulate-filled PCMs (low and medium filler content); wood–polymer composites
**>100**	**Non-polymeric materials:** paraffin; waxes; oily solid products **Polymers:** PE; PP; PMMA; PET (above T_g_); silicone resins **PCMs:** polymer–rubber composites; highly foamed polymer concretes; resin mortars; asphalt–polymer mixes

## Data Availability

The original contributions presented in this study are included in the article. Further inquiries can be directed to the corresponding author.

## Abbreviations

The following abbreviations are used in this manuscript:

ABS	acrylonitrile–butadiene–styrene copolymer
AH	asymptotic homogenization
BMIs	bismaleimide resins
CAB	cellulose acetate butyrate
CLT	classical laminate theory
CLTE	coefficient of linear thermal expansion
CMs	composite materials
CNTs	carbon nanotubes
CTE	coefficient of thermal expansion
CVTE	coefficient of volumetric thermal expansion
ECTFE	ethylene chlorotrifluoroethylene (copolymer)
EMT	effective medium theory
ERs	epoxy resins
ETFE	ethylene tetrafluoroethylene (copolymer)
FEP	fluorinated ethylene propylene (TFE–HFP copolymer)
HDPE	high-density polyethylene
LCP	liquid-crystal polymer
LDPE	low-density polyethylene
LVDT	linear variable differential transformer
MWCNTs	multi-walled nanotubes
PA6	polyamide 6 (nylon 6)
PA12	polyamide 12 (nylon 12)
PA66	polyamide 66 (nylon 66)
PAEK	polyaryletherketone (family)
PAI	polyamide-imide
PAR	polyarylate
PARA	polyarylamide (aromatic polyamide)
PBI	polybenzimidazole
PBT	polybutylene terephthalate
PC	polycarbonate
PCB	printed circuit board
PCMs	polymer composite materials
PCTFE	polychlorotrifluoroethylene
PDMS	polydimethylsiloxane
PE	polyethylene
PEI	polyetherimide
PEK	polyether ketone
PEKK	polyether ketone ketone
PEKEKK	poly(ether ketone ether ketone ketone)
PEEK	poly(ether ether ketone)
PEN	polyethylene naphthalate
PES	polyethersulfone
PFA	perfluoroalkoxy (TFE-based) copolymer
PFRs	phenol–formaldehyde resins
PET	poly(ethylene terephthalate)
PETG	glycol-modified poly(ethylene terephthalate)
PI	polyimide
PMMA	poly(methyl methacrylate)
PMP	poly(4-methyl-1-pentene)
PMPS	poly(methylphenylsiloxane)
POM	polyoxymethylene (acetal)
PP	polypropylene
PPA	polyphthalamide
PPO	poly(phenylene oxide)
PPS	poly(phenylene sulfide)
PPSU	poly(phenylsulfone)
PS	polystyrene
PSU	polysulfone
PTFE	polytetrafluoroethylene
PVDC	poly(vinylidene chloride)
PVDF	poly(vinylidene fluoride)
PVC	polyvinyl chloride
RVE	representative volume element
SAN	styrene–acrylonitrile copolymer
SWCNTs	single-walled nanotubes
T_g_	glass transition temperature
TMA	thermomechanical analysis
TPU	thermoplastic polyurethane
UHMW-PE	ultra-high-molecular-weight polyethylene
PPP	poly(p-phenylene)
